# Smoke Signals: Unraveling the Paradoxical Impact of Smoking on Stroke Prognosis and Treatment Efficacy

**DOI:** 10.7759/cureus.52346

**Published:** 2024-01-15

**Authors:** Faryal Zafar, Anesh Wadhwa, Kabeer Kumar, Muhammad Ahmed, Azhar S Khokhar, Sahar Sajjad, Sergio Rodrigo Oliveira Souza Lima, Shariq K Baluch, Abeer K Srour, Shadi S Al-Deir, Abdullah Shehryar, Abdur Rehman, Muhammad Abubakar

**Affiliations:** 1 Medicine, Ziauddin University, Karachi, PAK; 2 Medicine, Dow University of Health Sciences, Karachi, PAK; 3 Medicine and Surgery, Chandka Medical College, Larkana, PAK; 4 Psychiatry and Behavioral Sciences, Dow University of Health Sciences, Karachi, PAK; 5 Internal Medicine, Rasheed Nursing Home, Islamabad, PAK; 6 Medicine, Rasheed Nursing Home, Islamabad, PAK; 7 Plastic Surgery, Bahia Hospital, Salvador, BRA; 8 Internal Medicine, Universidad Autonoma de Guadalajara, Guadalajara, MEX; 9 Internal Medicine, Palestine Medical Complex, Ramallah, PSE; 10 Internal Medicine, Misr University for Science and Technology, Amman, JOR; 11 Internal Medicine, Allama Iqbal Medical College, Lahore, PAK; 12 Surgery, Mayo Hospital, Lahore, PAK; 13 Internal Medicine, Wah Medical College, Wah Cantt, PAK

**Keywords:** smoker's paradox, thrombolysis, post-stroke delirium, antiplatelet therapy, endovascular treatment, smoking, stroke prognosis

## Abstract

Smoking is a well-established risk factor for stroke, yet its impact on stroke prognosis remains complex and multifaceted. This systematic review aims to elucidate the relationship between smoking and various stroke outcomes, including response to treatment and long-term recovery.

We conducted a comprehensive analysis of four fundamental studies that examined the prognosis of stroke in smokers, focusing on clinical outcomes post-endovascular treatment, response to antiplatelet therapy, incidence of post-stroke delirium, and the effectiveness of thrombolysis treatment. The studies varied in design, including observational, retrospective, and post hoc trial analyses.

The review reveals that smoking may paradoxically predict better clinical outcomes in specific treatment scenarios, such as post-endovascular treatment and when using clopidogrel. However, smokers also demonstrated higher rates of ischemic stroke and post-stroke delirium. Notably, the *smoker's paradox* in thrombolysis treatment was not supported. These findings highlight the need for personalized treatment approaches based on smoking status.

Smoking has a complex and significant impact on stroke prognosis. While some benefits in specific treatment contexts were observed, the overall evidence strongly advises against smoking due to its adverse health consequences. This review underscores the importance of personalized stroke management in smokers and the integration of smoking cessation programs in post-stroke care. Future research should focus on larger, longitudinal studies to explore these associations further.

## Introduction and background

Stroke remains one of the leading causes of mortality and long-term disability worldwide, with an estimated 15 million individuals affected annually [1]. Despite advancements in prevention and treatment strategies, the global burden of this cerebrovascular disease continues to escalate, mainly due to modifiable lifestyle factors like smoking. Smoking, a well-established risk factor for stroke, not only increases the incidence but also significantly influences the prognosis and outcomes post-stroke [2,3].

The relationship between smoking and stroke prognosis is complex and multifaceted. Previous studies have shown that smoking can exacerbate the severity of stroke, influence response to treatment, and impact long-term recovery [4,5]. However, paradoxically, some research has also indicated potentially better outcomes in certain aspects of stroke recovery among smokers, a phenomenon often referred to as the *smoker's paradox* [6]. This dichotomy presents a compelling area for in-depth analysis, especially considering the global prevalence of smoking and the increasing incidence of stroke.

Given this backdrop, the objective of our systematic review is to critically evaluate and synthesize current research on the prognosis of stroke in smokers. We aim to dissect the nuances of how smoking influences various stroke outcomes, including response to treatment modalities, recovery rates, and long-term complications. By consolidating and analyzing data from multiple studies, this review seeks to provide a comprehensive understanding of the prognostic implications of smoking in stroke patients, thereby informing clinical practices and guiding future research in stroke management and prevention.

## Review

Materials and methods

Search Strategy

To systematically explore the prognosis of stroke in smokers, our search strategy was meticulously developed by PRISMA (Preferred Reporting Items for Systematic Reviews and Meta-Analyses) guidelines to encompass a wide array of pertinent literature. We initiated our search by querying several esteemed databases known for their comprehensive medical and scientific reports: PubMed, Embase, Web of Science, and Scopus. These databases were chosen for their extensive repositories of peer-reviewed articles, providing a solid foundation for our review.

The search was anchored by a carefully selected group of keywords and phrases relevant to our study objectives: "Stroke," "Cerebrovascular Accident," "Smoking," "Cigarette Use," "Prognosis," "Clinical Outcomes," "Endovascular Therapy," "Antiplatelet Therapy," and "Thrombolysis." Boolean operators "AND" and "OR" facilitated the creation of a multifaceted search algorithm. For example, we used the string "Stroke AND Smoking AND Prognosis" to yield studies explicitly examining the outcomes of stroke in the context of smoking. We also employed "OR" to encompass broader terms related to stroke and smoking, such as "Cerebrovascular Accident OR Stroke" and "Smoking OR Cigarette Use."

To ensure a contemporary and relevant scope, our search was restricted to studies published from the inception of each database to April 2023. This time frame allowed us to compile historical and cutting-edge research, offering a comprehensive overview. Additionally, we applied filters to include studies in English, human subjects, and adult populations to align with our review's focus.

The search was supplemented by manual searches of the reference lists of included studies and relevant reviews, ensuring that no pivotal study was overlooked. Our search strategy was both rigorous and expansive, aiming to capture the full spectrum of evidence regarding the impact of smoking on stroke prognosis. This structured approach provided a robust foundation for our systematic review, leading to an insightful synthesis of current knowledge in this critical area of medical research.

Eligibility Criteria

To establish a robust foundation for our systematic review of the prognosis of stroke in smokers, we meticulously delineated eligibility criteria that ensure precision and relevance in our research scope. We included peer-reviewed research articles, observational studies, and clinical trials, representing a wealth of high-quality, evidence-based knowledge. The population of interest for our review is adult patients, defined as individuals aged 18 or older who have suffered from a stroke, with a clear distinction in smoking status categorized as current smokers or nonsmokers at the time of the stroke event.

Our review encompasses explicitly studies that investigate poststroke medical interventions or examine the impact of smoking on stroke outcomes, including mortality, functional disability, recovery rates, and long-term complications. To guarantee the comprehensiveness and applicability of our findings, we have restricted our search to studies published in the English language, acknowledging its widespread use as the lingua franca of scientific communication. The time frame for inclusion extends from the inception of the respective databases to April 2023, thereby capturing the full spectrum of contemporary research.

Conversely, our exclusion criteria are tailored to uphold the focused nature of this review. Studies that do not directly address the relationship between smoking and stroke prognosis, such as those not differentiating outcomes based on smoking status or not examining post-stroke interventions, are omitted. To ensure the review's methodological rigor, we have excluded non-English language publications, unpublished works, and those considered gray literature, such as conference abstracts and posters. Additionally, studies that employ solely animal models or present insufficient data on the prognosis of stroke in smokers are excluded, maintaining the review's commitment to human-centered, data-rich research. Through these carefully crafted criteria, our review aspires to synthesize evidence that can reliably inform clinical practices and guide future investigations in stroke recovery and smoking.

Data Extraction

The data extraction phase of our systematic review was meticulously structured to uphold the integrity of our research findings. This critical process was executed in two distinct stages to ensure the thoroughness and accuracy of our data collection.

In the initial stage, we implemented a preliminary screening where articles were filtered based on the relevance indicated by their titles and abstracts. This phase was critical for establishing a foundational understanding of the study's relevance to our research question. Two independent reviewers, armed with expertise and an eye for detail, conducted this assessment, tagging each article as *relevant*, *not relevant*, or *probably relevant*. This classification was based on a conscientious appraisal of the study's abstract, gauging its pertinence to the nexus of smoking and stroke prognosis.

Progressing to the second stage, the full-text articles deemed *relevant* or *probably relevant* were subjected to a more granular review. Two independent reviewers delved into the detailed examination of these texts, employing a standardized data extraction template formulated within Microsoft Excel. This template was a uniform platform for meticulously capturing and organizing the data. Both reviewers independently applied our pre-established inclusion and exclusion criteria to each study, ensuring a consistent and unbiased selection process. Discrepancies between reviewers, albeit rare, were resolved through the adjudication of a third independent reviewer. This step was crucial for reaching a consensus and preserving the uniformity of the data extraction process.

The data extraction template was designed to capture an array of information critical to our systematic review, including but not limited to the author(s), year of publication, study's country of origin, participant demographics, study setting, design, methodologies, outcome measures, and critical findings. Such detailed data collation facilitated a nuanced synthesis and analysis, enabling us to draw meaningful conclusions from a holistic perspective. This rigorous data extraction process ensured that all relevant data points were captured, contributing to the robustness and credibility of our systematic review's conclusions.

Results

Study Selection Process

**Figure 1:**
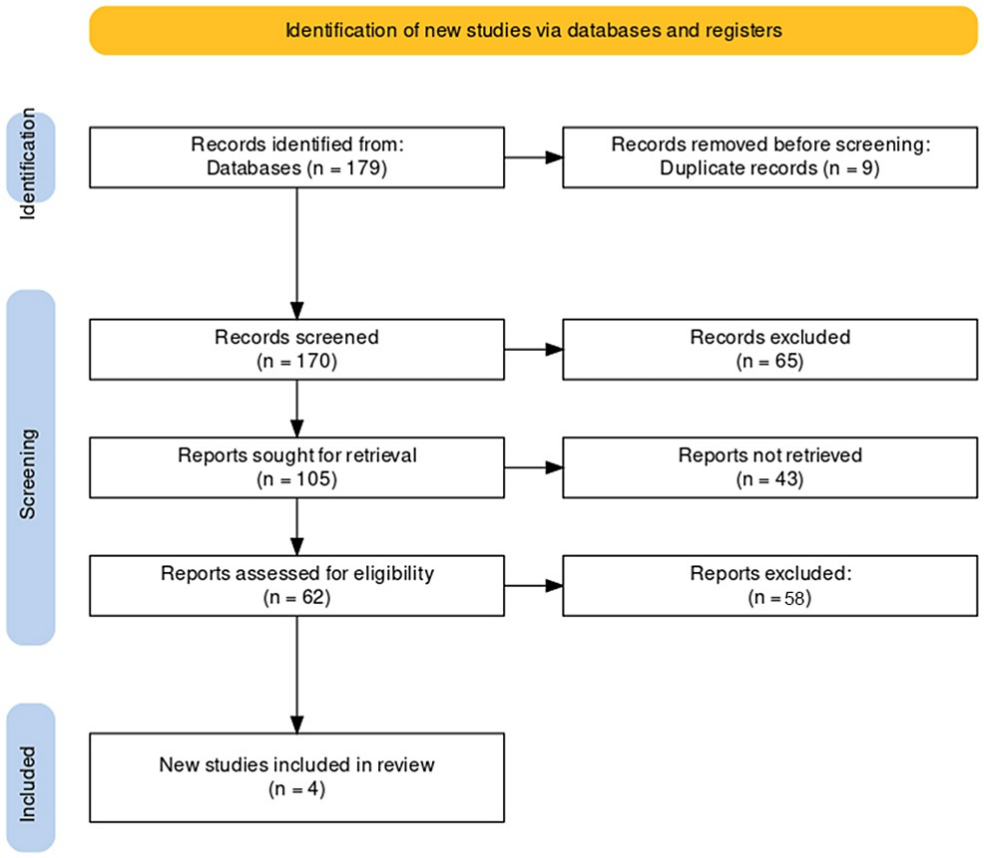
PRISMA flow diagram of the selection of studies for inclusion in the systematic review. PRISMA, Preferred Reporting Items for Systematic Reviews and Meta-Analyses

The study selection process for our systematic review of the prognosis of stroke in smokers was conducted with meticulous adherence to the PRISMA guidelines. Initially, a comprehensive search across various databases yielded 179 records. From these, we systematically removed nine duplicate records, thus refining our pool to 170 unique studies for screening. Each title and abstract were then carefully examined, excluding 65 records that did not meet our predefined relevance criteria. The remaining 105 articles underwent a more rigorous evaluation for eligibility, which involved retrieving and closely inspecting the full texts. This stage led to the exclusion of an additional 58 reports that failed to comply with our stringent inclusion criteria, either due to irrelevance to the specific focus on smokers' stroke prognosis or insufficient data quality. The culmination of this rigorous selection process resulted in four studies deemed suitable for inclusion in our review, providing a targeted and rich source of evidence for our subsequent analysis. The PRISMA flowchart is detailed in Figure 1.

Characteristics of Selected Studies

The four studies ultimately selected for inclusion in our systematic review were characterized by their focus on the prognostic outcomes of stroke in smoker populations. These studies, which spanned observational, retrospective, and post hoc trial analyses, encompassed a collective sample size of over 3,789 stroke patients, with subgroups delineating smokers from nonsmokers. The research originated from diverse geographical locations and healthcare settings, offering a broad perspective. They varied in design and scope but converged on examining the impact of smoking on poststroke clinical outcomes such as mortality, functional recovery, and secondary stroke incidence. The selected studies also explored the interaction between smoking and various stroke treatments, including endovascular therapy, antiplatelet therapy, and thrombolysis. This diversity in design and methodology provided a comprehensive cross-sectional view of the current understanding of how smoking affects stroke prognosis, thereby contributing significantly to our review's synthesis and analysis phase. The studies are outlined in Table 1.

**Table 1:** A summary of studies included in this systematic review.

Title	Authors	Year	Study design	Sample size	Key findings	Conclusions	Level of evidence
Impact of Smoking on Stroke Outcome After Endovascular Treatment [7]	von Martial et al.	2018	Observational study	935 stroke patients (204 smokers, 731 nonsmokers)	- Higher rates of excellent clinical outcomes in smokers - Lower mortality in smokers - No difference in risk of symptomatic intracranial hemorrhage (sICH)	Smoking may predict better outcomes after endovascular treatment (EVT) but should not be considered beneficial.	Level II (Observational study)
Clopidogrel and Ischemic Stroke Outcomes by Smoking Status: Smoker’s Paradox? [8]	Zhang et al.	2016	Retrospective single-center study	1,792 ischemic stroke patients (1,066 smokers, 726 never-smokers)	- Higher stroke rates in smokers - Lower vascular event rates with clopidogrel vs. aspirin in smokers	Smoking increases recurrent stroke risk. Clopidogrel may lead to better outcomes for smokers	Level II (Retrospective study)
Cigarette Smoking Is an Independent Risk Factor for Post-stroke Delirium [9]	Lim et al.	2017	Retrospective analysis	576 ischemic stroke patients	- Higher delirium rates in smokers (40%) than nonsmokers (24%) - Smoking an independent delirium risk factor	Smoking may increase poststroke delirium risk, which needs more research.	Level II (Retrospective study)
Current Smoking Does Not Modify the Treatment Effect of Intravenous Thrombolysis in Acute Ischemic Stroke Patients—A Post-hoc Analysis of the WAKE-UP Trial [10]	Schlemm et al.	2019	Post hoc analysis of WAKE-UP trial	486 acute ischemic stroke patients	- No impact of smoking on alteplase treatment effect - Worse outcomes in smokers	Smoking does not alter alteplase effectiveness in stroke	Level II-III (Post hoc analysis of a clinical trial)

Discussion

This systematic review critically examines the multifaceted impact of smoking on the prognosis of stroke, encompassing various outcomes and treatment responses. The studies analyzed present a paradoxical narrative, where smoking, a known risk factor for stroke, appears to influence poststroke outcomes and treatment efficacy in complex and sometimes seemingly contradictory ways.

In our comprehensive review, we have observed that the smoking status of stroke patients substantially affects the effectiveness of treatment modalities and the trajectory of recovery following a stroke. Notably, the study conducted by von Martial et al. [7] provides evidence suggesting that smokers may paradoxically exhibit more favorable clinical outcomes following endovascular treatment (EVT) when compared to their nonsmoking counterparts. This finding is particularly intriguing as it posits a seemingly advantageous impact of smoking in the context of specific poststroke interventions.

However, it is imperative to approach these findings with a nuanced understanding. The apparent better outcomes in smokers post-EVT should not be interpreted as a vindication of smoking. In reality, the broader and more well-established body of scientific literature consistently identifies smoking as a significant risk factor for stroke and a host of other cardiovascular diseases [2,3]. The deleterious effects of smoking on vascular health are well-documented, encompassing the exacerbation of atherosclerosis, increased clotting propensity, and overall detriment to cardiovascular integrity.

Thus, while the study by von Martial et al. [7] contributes valuable insights into the complex interplay between smoking and stroke recovery, particularly in the context of EVT, these findings must be contextualized within the larger framework of smoking's overall impact on health. This underscores the need for a careful and comprehensive approach to interpreting the implications of smoking on stroke prognosis, balancing the paradoxical findings with the broader understanding of smoking as a major health hazard.

Therefore, the phenomenon observed in the study should be viewed as a potential avenue for further investigation rather than as a rationale for mitigating the well-established public health message against smoking. Future research endeavors should aim to elucidate the mechanisms underlying these paradoxical outcomes, potentially uncovering novel aspects of stroke pathology and recovery that could inform more effective and personalized treatment strategies for stroke patients.

In line with this, the differential response to antiplatelet therapy based on smoking status, as observed by Zhang et al. [8], suggests a potential pharmacodynamic interaction between smoking and clopidogrel. Smokers on clopidogrel therapy demonstrated a lower rate of composite vascular events, highlighting the need for personalized treatment strategies in stroke management [11].

Our findings challenge the notion of the *smoker's paradox*. While some studies have suggested that smoking may confer a protective effect in specific acute ischemic stroke (AIS) treatment scenarios, our analysis, including the post hoc analysis of the WAKE-UP trial data by Schlemm et al. [10], does not support this hypothesis. It underlines the importance of interpreting such paradoxical findings cautiously and stresses the need for robust, prospective research to clarify these associations.

The research conducted by Lim et al. [9] significantly contributes to our understanding of smoking's impact on stroke recovery by highlighting the association between smoking and the increased incidence of poststroke delirium. This finding adds a crucial layer to the multifaceted public health challenge posed by smoking. Delirium after stroke, characterized by acute cognitive dysfunction, disorientation, and reduced awareness, significantly complicates the recovery process, potentially leading to prolonged hospital stays, increased healthcare costs, and a higher likelihood of long-term cognitive impairment. The association between smoking and this acute neuropsychiatric syndrome emphasizes how smoking not only contributes to the initial risk of stroke but also to post-stroke complications that can severely hinder recovery and rehabilitation efforts.

Given these implications, the integration of smoking cessation strategies into poststroke care becomes paramount. By addressing smoking as a modifiable risk factor, healthcare providers can significantly influence the trajectory of stroke recovery. Evidence suggests that smoking cessation can markedly reduce the risk of primary stroke [4], and this preventative benefit extends to the poststroke phase, where it plays a critical role in mitigating secondary complications like delirium [12,13]. The implementation of targeted smoking cessation programs in stroke rehabilitation settings could, therefore, be a critical step in enhancing patient outcomes. These programs should be tailored to address the unique challenges faced by stroke survivors, incorporating comprehensive support mechanisms that include counseling, pharmacotherapy, and continuous follow-up. Such an integrated approach promises to improve individual patient outcomes and contributes to broader public health efforts to reduce the burden of stroke and its associated complications.

While our review provides significant insights, it also highlights the need for further research. The retrospective nature and the variability in methodologies of the included studies necessitate a cautious interpretation of the findings. Future research should aim for larger sample sizes, more extended follow-up periods, and comprehensive data on smoking habits, including the impact of smoking cessation post-stroke.

This systematic review meticulously elucidates the intricate and consequential effects of smoking on stroke prognosis and the efficacy of its treatment modalities [14]. The complexity of these effects is underscored by certain studies suggesting a *smoker’s paradox*, where smoking appears to confer some level of protective benefit in specific treatment scenarios, such as improved outcomes post-EVT or response to certain pharmacological interventions. However, it is critical to contextualize these findings within the broader spectrum of evidence that overwhelmingly delineates smoking as a major risk factor for stroke and a host of other detrimental health outcomes [15]. The adverse effects of smoking are well-documented and multifarious, ranging from the exacerbation of atherosclerosis and increased propensity for thrombosis to the impairment of vascular function and overall cardiovascular health.

In light of this, the review strongly advocates for a healthcare approach that not only acknowledges the nuanced impacts of smoking on stroke treatment and prognosis but also vigorously promotes smoking cessation as a cornerstone of stroke management and recovery. The development and implementation of personalized treatment strategies that take into account an individual's smoking history and status are imperative. Such strategies should encompass a comprehensive plan that not only addresses the acute management of stroke but also actively incorporates smoking cessation programs as an integral part of post-stroke care. These programs, tailored to the needs and circumstances of each patient, could significantly enhance recovery outcomes by reducing the risk of recurrent strokes and other smoking-related complications.

Because of the valuable insights gained from this systematic review, it is essential to acknowledge certain limitations that may affect the generalizability and interpretation of our findings. First, our review was confined to studies published in English, which may have excluded relevant research conducted in other languages, potentially introducing a selection bias. Second, the inclusion of only four studies in our final analysis, with varying methodologies and designs (observational, retrospective, and post hoc analyses), raises concerns regarding the breadth and uniformity of the data. Such diversity in study designs could impact the comparability of results and limit our ability to draw broad, generalizable conclusions. Moreover, the retrospective nature of some included studies might introduce inherent biases, affecting the reliability of the outcomes. The absence of longitudinal data and limited information on the duration and intensity of smoking habits in the reviewed studies also constrains the depth of our conclusions, particularly concerning the long-term implications of smoking on stroke prognosis. Future research addressing these gaps, with more extensive and diverse study designs, is crucial for a more comprehensive understanding of the complex interplay between smoking and stroke outcomes. Acknowledging these limitations is vital for a balanced interpretation of our findings and their application in clinical and public health contexts.

## Conclusions

Our systematic review reveals a complex relationship between smoking and stroke prognosis, challenging the conventional understanding of smoking's role in poststroke outcomes. While smokers may show unexpected benefits in specific treatment responses, such as endovascular therapy and clopidogrel, these findings should not overshadow the established risks of smoking, including its significant contribution to stroke incidence and overall cardiovascular risk. This paradox underscores the necessity for personalized treatment strategies in stroke care, particularly considering the smoker's status, and highlights the urgent need for integrating smoking cessation programs in poststroke rehabilitation to improve long-term recovery and quality of life.

The implications of our review are clear: While certain aspects of smoking in stroke prognosis may appear protective in specific contexts, the overall evidence firmly supports the detrimental effects of smoking. Future research should aim for comprehensive studies with more extensive and diverse populations to further unravel the impact of smoking cessation on stroke prognosis. Ultimately, our findings advocate for a nuanced approach to managing stroke in smokers and reinforce the critical public health message of smoking cessation as a cornerstone for stroke prevention and enhanced recovery outcomes.

## References

[1] Johnson W, Onuma O, Owolabi M, Sachdev S (2016). Stroke: a global response is needed. Bull World Health Organ.

[2] O’Donnell MJ, Chin SL, Rangarajan S (2016). Global and regional effects of potentially modifiable risk factors associated with acute stroke in 32 countries (INTERSTROKE): a case-control study. Lancet Lond Engl.

[3] Hackam DG, Mrkobrada M (2012). Selective serotonin reuptake inhibitors and brain hemorrhage: a meta-analysis. Neurology.

[4] Shah RS, Cole JW (2010). Smoking and stroke: the more you smoke the more you stroke. Expert Rev Cardiovasc Ther.

[5] West R (2017). Tobacco smoking: health impact, prevalence, correlates and interventions. Psychol Health.

[6] Lim KH, Cheong YL, Lim HL (2022). Assessment of association between smoking and all-cause mortality among Malaysian adult population: findings from a retrospective cohort study. Tob Induc Dis.

[7] von Martial R, Gralla J, Mordasini P (2018). Impact of smoking on stroke outcome after endovascular treatment. PLoS One.

[8] Zhang Q, Wang Y, Song H (2017). Clopidogrel and ischemic stroke outcomes by smoking status: smoker's paradox?. J Neurol Sci.

[9] Lim TS, Lee JS, Yoon JH, Moon SY, Joo IS, Huh K, Hong JM (2017). Cigarette smoking is an independent risk factor for post-stroke delirium. BMC Neurol.

[10] Schlemm L, Kufner A, Boutitie F (2019). Current smoking does not modify the treatment effect of intravenous thrombolysis in acute ischemic stroke patients-a post-hoc analysis of the WAKE-UP trial. Front Neurol.

[11] Sibbald M, Yan AT, Huang W (2010). Association between smoking, outcomes, and early clopidogrel use in patients with acute coronary syndrome: insights from the Global Registry of Acute Coronary Events. Am Heart J.

[12] Zhang P, Guo ZN, Sun X, Zhao Y, Yang Y (2019). Meta-analysis of the smoker’s paradox in acute ischemic stroke patients receiving intravenous thrombolysis or endovascular treatment. Nicotine Tob Res.

[13] Iglesias-Rey R, Custodia A, Alonso-Alonso ML (2022). The smoking paradox in stroke patients under reperfusion treatment is associated with endothelial dysfunction. Front Neurol.

[14] Luo J, Tang X, Li F (2021). Cigarette smoking and risk of different pathologic types of stroke: a systematic review and dose-response meta-analysis. Front Neurol.

[15] Matsuo R, Ago T, Kiyuna F (2020). Smoking status and functional outcomes after acute ischemic stroke. Stroke.

